# Angina Paralytica in the Horse Caused by the Use of Ensilage*Giornale di Anal. Fisiol. e Patol. degli Animali.

**Published:** 1885-01

**Authors:** Mario Ortolani


### IV.—ANGINA PARALITICA IN THE HORSE CAUSED BY THE USE OF ENSILAGE.*

BY DR. MARIO ORTOLANI.

Through the experiments made by Baron Turriri on his farm, the practice has spread through Sicily of storing in silos the fodder which grows spontaneously in the natural Sicilian meadows.

It now appears that two proprietors who followed the example of the Baron, giving this ensilage to their horses, have seen them first become sick, and afterwards die with symptoms described in the reports which are attached.

Meanwhile it is to be remarked that the feeding of cattle here in Palermo with two-thirds of ensilage, composed principally of *Avena Sterilis, L*, the other third being composed of different vegetable matter, has given excellent results, and I have not as yet to lament any deterioration in the health of the cattle.

Called to study the cause of death in the horses, the first idea which came to my head was that some fungus in the fodder was the cause of the pathological changes in the mucous membrane of the nose, mouth, and pharynx, but was unable to make microscopical investigation. For the present I will limit myself to a reference to the reports of the proprietors who suffered loss.

The first states that until the twelfth day of the administration of the fodder, the animals thrived. Then it was noticed that, although with desire for food and ability to masticate, they were unable to swallow solids or liquids. The tongue became paralyzed, and hung from the mouth. On the following morning the sight was lost, and in twenty-four hours from the first manifestation of disease death took place. Autopsy disclosed ulceration of the pharynx as the only abnormal condition. None of the animals had fever, and there were no diphtheritic patches.

I examined some of the fodder administered in this case, but found nothing to call my attention except that on some grains of wheat were found bacteria, which in our cereals produces inflammation of the mucous membrane of the mouth, eyes, etc., accompanied by fever. I fed my own cattle on the same fodder, and no bad result followed.

The second reports that the animals fed on the ensilage did well until the twelfth day, when they were attacked in a similar manner to those in the preceding account, death carrying off the majority of them.

I have used various kinds of ensilage by way of experiment, and thirty of my cattle have fed thereon during six months, with the best results.

---

* Giornale di Anal. Fisiol. e Patol. degli Animali.

